# Fast ion transport through ultrathin shells of metal sulfide hollow nanocolloids used for high-performance energy storage

**DOI:** 10.1038/s41598-017-18504-6

**Published:** 2018-01-08

**Authors:** Zhenhua Chen, Mengen Zhao, Xinyan Lv, Kang Zhou, Xiaoqian Jiang, Xiuli Ren, Xifan Mei

**Affiliations:** Jinzhou Medical University, Jinzhou, 121001 People’s Republic of China

## Abstract

Metal sulfide (MS, nickel sulfide/copper sulfide) hollow spheres with hierarchical, ultrathin shell structures have been constructed by a facile method. The as-formed MS hollow structures are shown to be uniform in sizes with hierarchical ultrathin shells, which could facilitate the transport of electrolyte ions. Electrochemical evaluations of the as-fabricated MS based materials as supercapacitors electrodes having high large surface area (106–124 m^2^ g^−1^) and high specific capacitances (up to 1460 F g^−1^) with good cycling stability (up to 94% retention after 5000 cycles), showing their potential applications in the next-generation high-performance supercapacitors used for energy storage.

## Introduction

The global climate change with increasing environmental issues in recent years have inspired great efforts on exploring sustainable and renewable energy, such as solar and nuclear energy^1–3^. As such, high-performance electrochemical energy storage devices such as lithium-ion/sodium ion batteries and supercapacitors are indispensable to store and utilize the above-mentioned energy source^4–6^. As known to all, supercapacitors are widely used alternative power supply, which deliver higher power density and longer cycle life compared to their battery counterparts^7–10^. Thus, the supercapacitors are seen to play a more important role for upcoming large-scale applications such as electric vehicles and hybrid electric vehicles^11^.

Exploration of suitable electrode materials is essential to develop high-performance supercapacitors. The active carbon materials are one of the popular candidates for supercapacitors owing to their low cost, high chemical stability and controllable porosity^12,13^. However, the relatively low specific capacitances or energy densities of various carbon materials reported in the past few years have shown their limitation in future practical applications. To address this issue, the research community has paid increasing attentions to transition metal oxides (TMO) or sulfides (TMS) materials, which can deliver much higher specific capacitance because of their high electroactivity^14–23^. For example, a high specific capacitance of 1370 F g^−1^ can be achieved at a current density of 2 A g^−1^ for 3D Ni_3_S_2_nanosheets in a recent report^24^. In another work by Liu *et al*., NiCo_2_S_4_ nanostructured arrays on carbon fiber papers exhibited a specific capacitance of 1154 F g^−1^ at a current density of 1 A g^−1^ with excellent cycling life^25^. Hence, the intrinsic redox characteristics of the TMS endow the according electrodes more promising in supercapacitors, which work in both non-faradaic (double layer capacitance) and faradaic regions (battery-like pseudo capacitance), thus further boosting the over-all specific capacitances^26^.

An efficient strategy to make a better use of the electroactivity of TMS is to fabricate hollow nanostructures, which largely increases active sites of the electrodes by offering large surface area (SSA) and improving electrolyte ions transport^27–29^. For example, Wei and co-workers have developed an efficient ethanol thermal reduction method to prepare hierarchical ZnV_2_O_4_ microspheres and MoO_2_-C hollow spheres for enhanced electrochemical performances^30,31^. Meanwhile, it has been well proved that hollow structures with hierarchical shells based on TMS usually exhibit enhanced electrochemical performances by virtue of electrode/electrolyte interface charge transfer^32–37^. However, despite the recent advances in preparation of various TMS materials with hollow structures, it is still a big challenge to develop an effective general approach for achieving free-standing TMS hollow spheres with uniform size distributions.

Herein, we reported an effective general method to prepare MS hollow spheres *via* a facile template-engaged method. This synthetic strategy involves the template-engaged deposition of hierarchical precursor shells and a subsequent sulfurization process. The silica colloids (SC) spheres were employed as hard template for the first hydrothermal deposition of metal precursor (MP) in the presence of urea. Two different types of MP were obtained at this stage, which are nickel based (MP-Ni) nanosheets and copper based (MP-Cu) nanoneedles. The as-obtained MP was then converted to corresponding MS hydrothermally in the presence of thiourea. Simultaneously, the SC templates were removed during this sulfurization process, leading to the formation of MS hollow structures (MS-Ni and MS-Cu).Owing to these compositional and structural features, the as-constructed MS hollow nanocolloids shave demonstrated high specific capacitances with good cycling stabilities when employed as electrode materials for supercapacitors.

## Experimental

### Synthesis of SC@MP

To prepare SC@MP-Ni, 36 mg of SiO_2_ (400 nm) was dispersed into 40 mL of DI water by ultrasonication for 10 min, followed by the addition of 0.72 g of urea. After 5 min, 0.5 mL of Ni(NO_3_)_2_ aqueous solution (0.12 M) was added, and the mixture was sealed in a blue-cap glass bottle and heated at 105 °C for 9 h. After cooling down to room temperature, the green products were harvested by several rinse-centrifugation cycles and fully dried at 60 °C for further use at the next step. The SC@MP-Cu was also synthesized by a similar procedure, but 0.1 mL of concentrated ammonia solution was added instead of urea, and 0.6 mL of Cu(NO_3_)_2_ aqueous solution (0.12 M) was added as copper source.

### Synthesis of MS hollow structures

For the preparation of MS hollow spheres, 15 mg of the as-prepared SC@MP (SC@MP-Ni and SC@MP-Cu) was dispersed into 30 mL water/ethanol (ethanol v% = 50%) by ultrasonication for 10 min, followed by the addition of 50 mg of thiourea. After 5 min, the mixture was sealed in a blue-cap glass bottle and then heated at 120 °C for 6 h. The products were allowed to cool down to room temperature naturally, and collected by the rinse-centrifugation process with DI water and ethanol several times. The obtained products were thoroughly dried at 60 °C in vacuum for further characterization and usage. A similar strategy was employed to synthesize worm-like hollow nanorods (both Ni and Cu cases) following the same synthesis process with MS hollow spheres.

### Material characterizations

All the samples were characterized by field-emission scanning electron microscopy (FESEM, JEOL, JSM-6304F) equipped with an energy dispersive X-ray spectroscopy (EDX), transmission electron microscopy (TEM, JEOL, JEM-2010) and X-ray diffraction (XRD, Bruker, D8-Advance Diffractometer, Cu Ka). The BET properties of the MS samples were carried out using a N_2_ adsorption-desorption at 77 K with a Quantachrome NOVA-3000 system.

### Electrochemical measurements

The capacitor electrodes were fabricated by mixing the active materials with carbon black (super-P) and polyvinylidenedifuoride (PVDF) at a weight ratio of 8:1:1. After thorough mixing by a magnetic stirring, the slurry was pressed onto a piece of Ni foam (1*3 cm) and was dried at 60 °C in vacuum for 12 h. The mass loading of the active materials is ~2 mg for each electrode. The electrochemical measurements were conducted with a CHI 660E electrochemical workstation in an aqueous KOH electrolyte (1 M) with a three-electrode system, where a Pt foil served as the counter electrode and a standard calomel electrode (SCE) as the reference electrode. The as-prepared MS-Ni and MS-Cu hollow spheres were also used as electrode materials for fabricating asymmetrical supercapacitors (ASC, MS-Ni ‖ MS-Cu) following a similar preparation method for working electrodes for three-electrode test. The electrochemical measurements were conducted in 1 M KOH aqueous solution and the specific capacitances were calculated based on the total mass of the two electrodes.

## Results and Discussion

The synthetic strategy for MS hollow spheres is schematically illustrated in Fig. S1. At first, SC (D~400 nm) spheres are employed as templates for the uniform deposition of MP, namely MP-Ni nanosheets and MP-Cu nanoneedles by a hydrothermal synthesis in the presence of urea or ammonia (see experimental details), forming the SC@MP core-shell structures (step I). The urea herein has played a role as a surfactant for the formation of MP, which activated the SC surface to facilitate the reactions between the metal salts and SC. The as-formed MPs are then subsequently converted to MSs through a similar hydrothermal process by introducing thiourea into the synthesis as a sulfur source (step II). As a result, the MPs are completely sulfurized into MS with their hierarchical structures well preserved. Meanwhile, the SC templates have been eliminated during the hydroxyl group-rich sulfurization process, thus generating hollow interiors of the MS particles.

**Figure 1:**
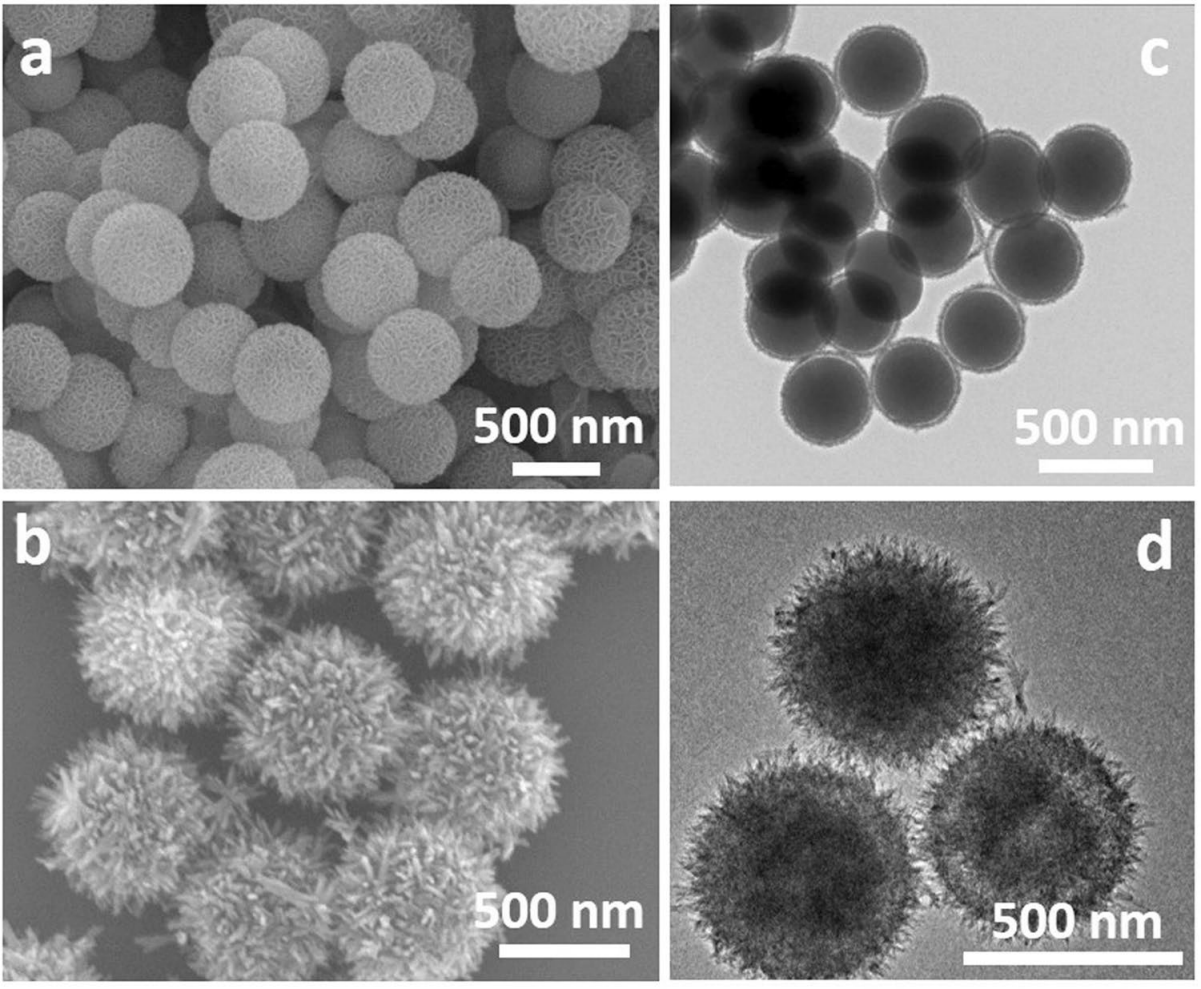
FESEM (**a** and **b**) and TEM (**c** and **d**) images of the as-prepared MP-Ni (**a** and **c**) and MP-Cu (**b** and **d**).

The uniform deposition of MP’s hierarchical nanostructures on SC templates is confirmed by the field-emission scanning electron microscopy (FESEM) images as displayed in Fig. 1a (MP-Ni) and Fig. 1b (MP-Cu). Both MP-Ni and MP-Cu nanoparticles are uniform in size (D = ~420 nm for MP-Ni and ~480 nm for MP-Cu) and no aggregation is observed. Transmission electron microscopy (TEM) images (Fig. 1c,d) further reveal the details of the MP structures, where obvious core-shell structures and hierarchical hairy shells can be observed for MP-Ni (Fig. 1c) and MP-Cu (Fig. 1d), respectively. It also can be seen from the TEM results that the SC templates in both of the MP samples are still retained at this stage (step I in Fig. S1), which has confirmed a template-engaged synthesis in this strategy. Energy dispersive X-ray (EDX) was carried out to find out the elements presented in the MP samples and the results are shown in Fig. S2. Peaks of Ni, Cu, Si and O are present in these MP samples (Fig. S2a and b). This indicates the formation of metal-Si precursors. However, no strong X-ray diffraction (XRD) peaks can be picked out for both of the MP samples, showing the amorphous characteristics of the MP (Fig. S2c and d).

**Figure 2:**
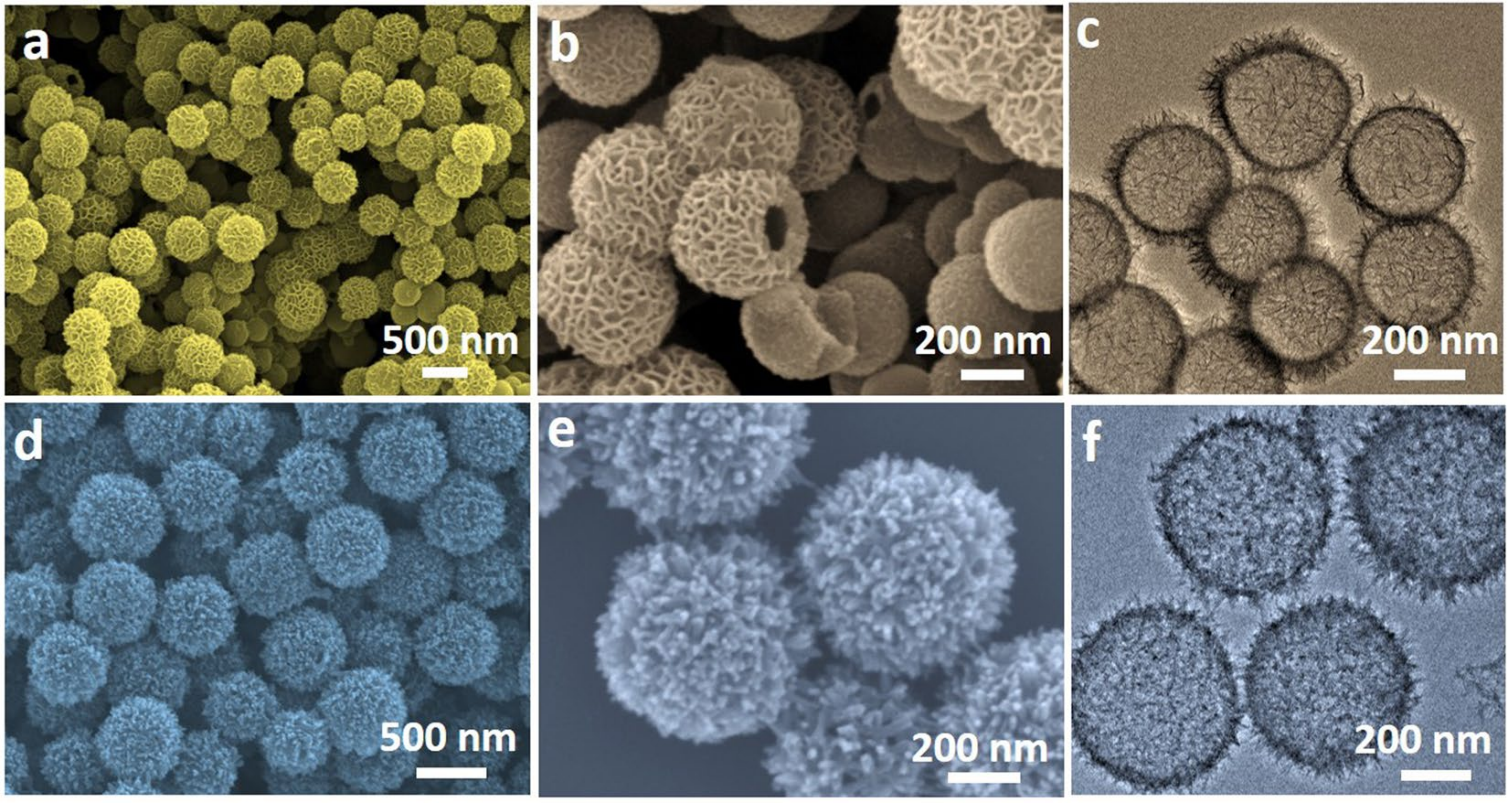
FESEM (**a**,**b**,**d** and **e**), TEM (**c** and **f**) images of the as-prepared MS-Ni (**a**–**c**) and MS-Cu (**d**–**f**) hollow nanostructures.

**Figure 3:**
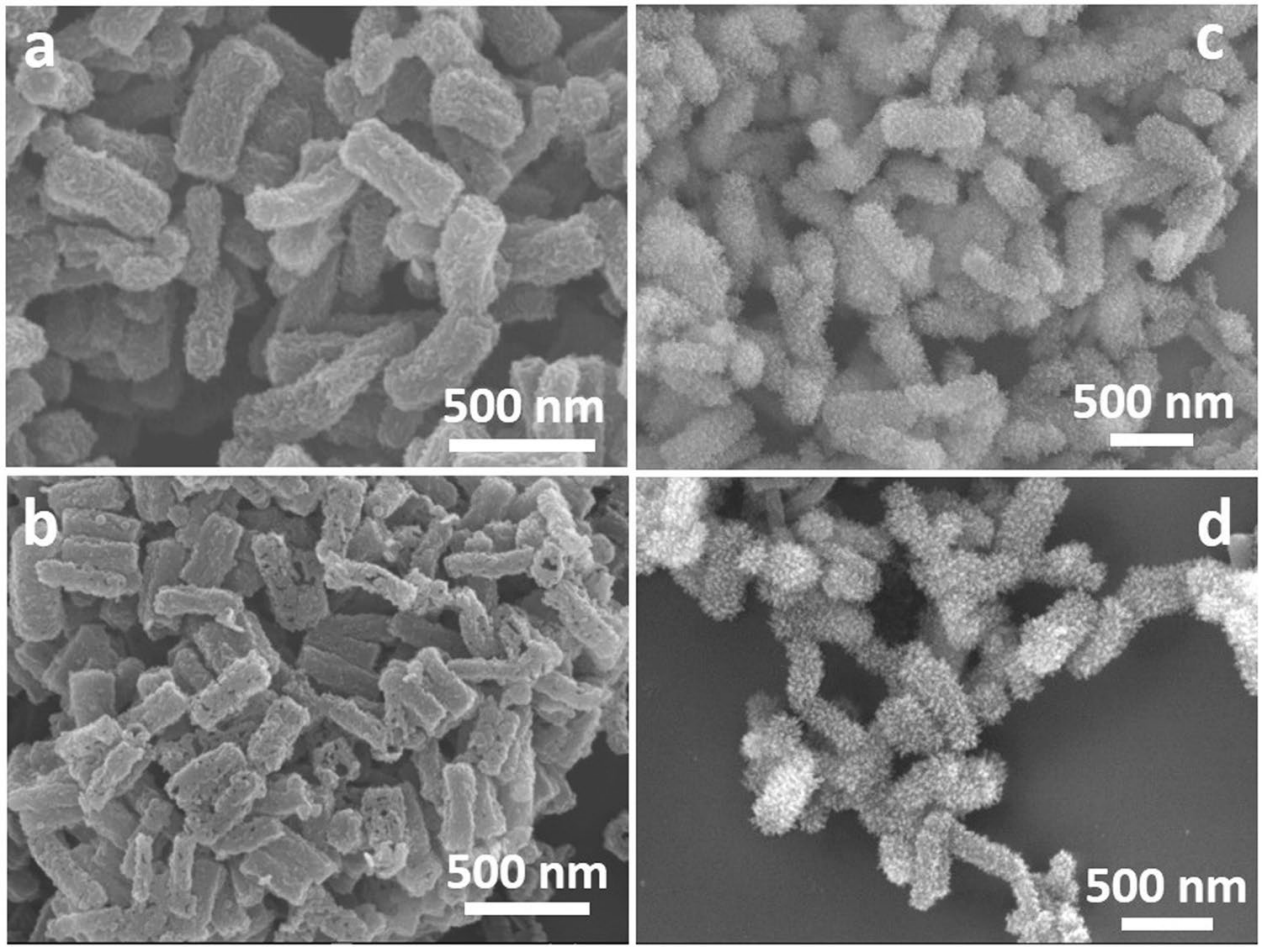
FESEM images of the worm-like MP-Ni (**a**), MP-Cu (**c**), corresponding MS-Ni (**b**) and MS-Cu (**d**) hollow nanostructures.

The as-formed MP@SC core-shell structures were then converted to corresponding MS hollow spheres by a subsequent hydrothermal sulfurization. Typical FESEM and TEM images of the MS products are displayed in Fig. 2. It can be seen that the spherical shapes with the nanosheet shells of the MS-Ni particles are well reserved after sulfurization, revealing the structural robustness of the particles (Fig. 2a). The hollow interiors of MS-Ni can be observed by some of the broken individual particles (Fig. 2b). Furthermore, the hollow structures of MS-Ni can be reflected by a TEM image in Fig. 2c, from which hollow spheres with hierarchical shells (10–20 nm in thickness) can be clearly observed. For the MS-Cu sample, the hairy structures of the spheres are well retained despite the nanoneedles of the shells have been shortened after sulfurization (Fig. 2d,e). Compared to the MP-Cu samples (Fig. 1b,d), the MS-Cu particles have been hollowed after sulfurization, generating a chestnut-like hollow structure with an average shell thickness of 10 nm (Fig. 2f). The compositional ratios of all the elements in the hollow nanospheres were determined by the EDX analysis (Fig. S3). The percentages of Ni and S in the MS-Ni hollow spheres (I, Fig. S3) are 62.4% and 36.2%, respectively, while the percentages of Cu and S in the MS-Cu hollow spheres ((III, Fig. S3) are 56.8% and 42.2%, respectively. The crystallographic phase of the as-prepared MS products was examined by XRD (Fig. S4). All the diffraction peaks can be assigned to Ni_3_S_2_ phase (JCPDS NO. 44-1418)^38^, indicating the complete conversion from amorphous MP-Ni to pure Ni_3_S_2_. Different from the MS-Ni case, a mixed phase of CuS (JCPDS NO. 06-0464)^39^ and Cu_1.8_S (JCPDS NO. 72-1996)^40^ can be found for the MS-Cu hollow spheres. The multi-valence states could be favorable to the redox reactions occurred in the electrochemical process of the electrodes. Interestingly, this synthetic strategy can be extended to prepare worm-like MS hollow structures when using silica nanorods as hard templates. MS-Ni in nanosheets (Ni:S = 66.3%:31.8%, II of Fig. S3) and MS-Cu in nanoneedles (Cu:S = 58.5%:38.7%, IV of Fig. S3) have been fabricated following a similar chemical process, which shows the generality of this synthetic methodology (Fig. 3).

**Figure 4:**
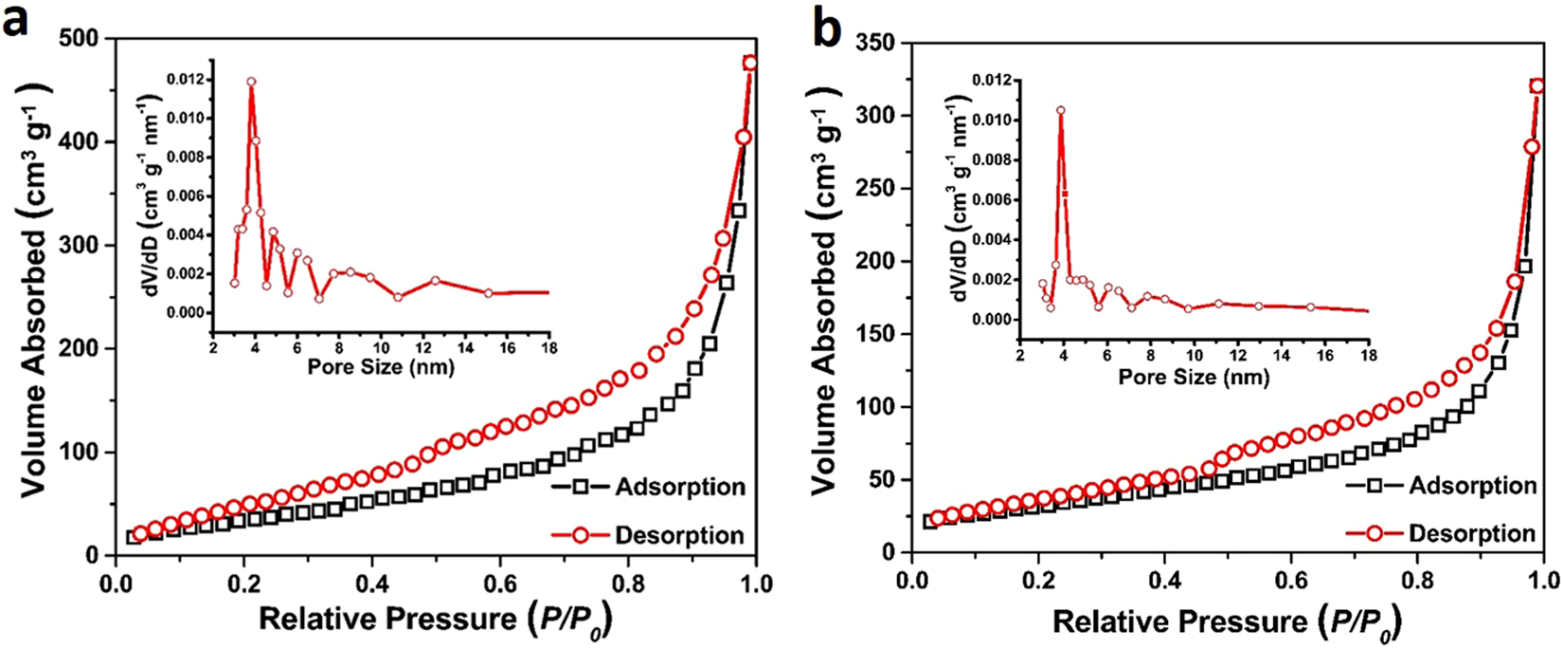
Nitrogen adsorption-desorption isotherms of MS-Ni (**a**) and MS-Cu (**b**). The insets are the corresponding pore size distributions.

Owing to the hierarchical shell and large voids, high SSA may be endowed for these MS hollow spheres. Thus, the Brunauer-Emmett-Teller (BET) measurements were carried out to study the porosity and pore size distribution of the MS samples. SSA of *ca*. 124 m^2^ g^−1^ is achieved for sample MS-Ni with an average pore size of 3.5 nm (Fig. 4a). The MS-Cu sample delivers a BET specific surface area of *ca*. 106 m^2^ g^−1^ with a larger average pore size of 4.2 nm (Fig. 4b). Apparently, such hierarchical hollow structures with ultrathin shells and high SSA are anticipated to offer tremendous active sites for electrochemical reactions and facilitate the electrolyte ion transport through the shell structures. In addition, the hollow interiors could also serve as a reservoir for electrolyte, which can improve the diffusion of electrolyte ions at the interface between the electrode and electrolyte^41^.

As promising electroactive materials, both of the MS hollow spheres were employed as electrode materials for hybrid supercapacitors. At first, electrochemical surface areas (ECSA) were estimated from non-Faradaic cyclic voltammetry (CV) curves to evaluate the true surface of the electrodes (Fig. S5). CV measurements were performed within a same potential range of 0 to 0.1 V (vs. SCE) at different scan rates (5–60 mV s^−1^) for MS-Ni (Fig. S5a) and MS-Cu (Fig. S5b), respectively. The capacitances of the two samples calculated from the above CV curves are shown in Fig. S5c, where values of 1.36 and 1.21 mF are delivered for MS-Ni and MS-Cu, respectively. In this case, higher capacitance denotes a higher ECSA because the ECSA is proportional to the capacitance according to the following equation:

1 $$C={\boldsymbol{\varepsilon }}S/4\pi kd$$

where C is the capacitance, ε is the dielectric constant, S is the ECSA, k is the Boltzmann constant and d is the distance between the two double layers. Thus, a higher ECSA may lead to a better electrochemical performance when referring to a same electrode material.

**Figure 5:**
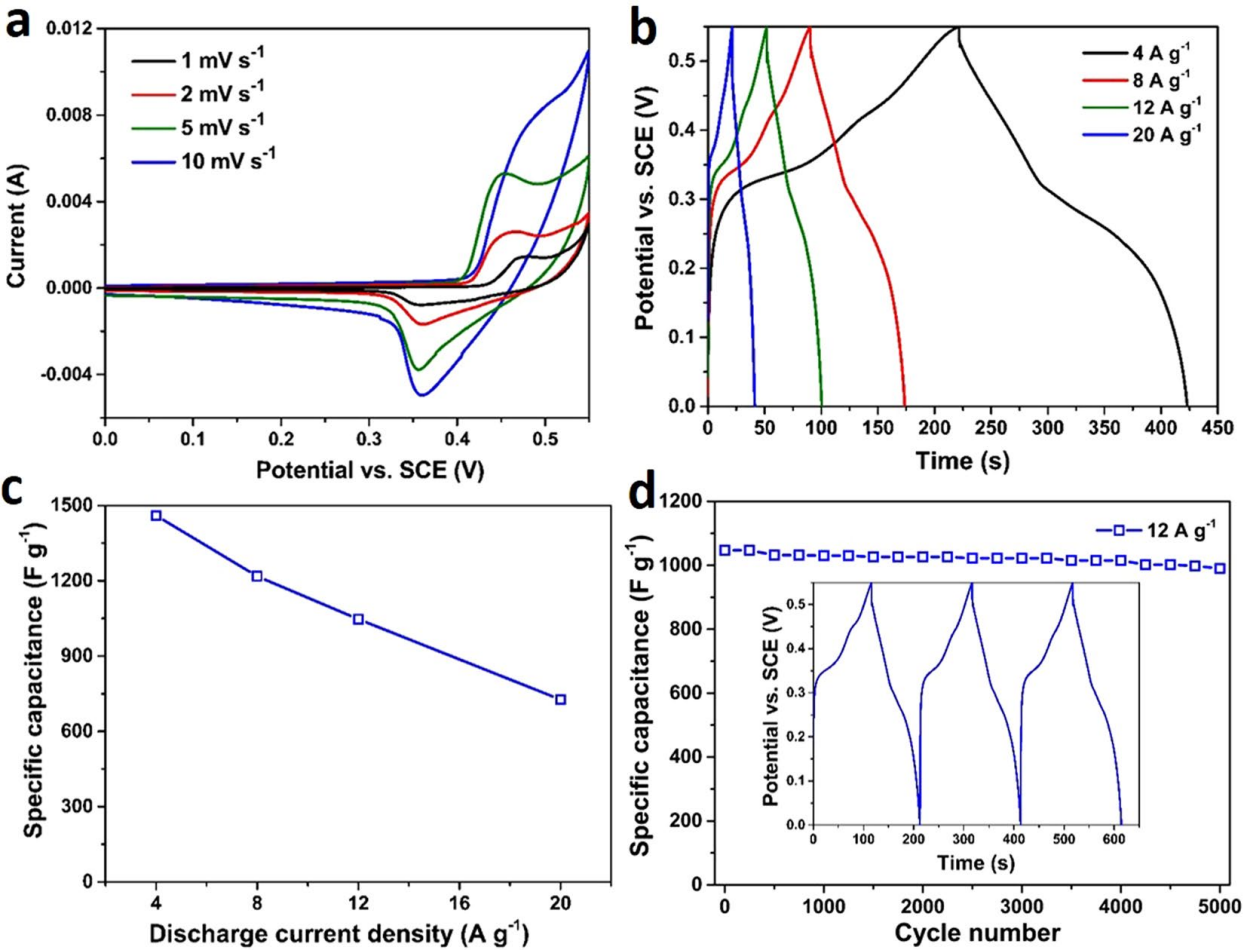
CV curves (**a**), GCCD curves (**b**), specific capacitances calculated from different discharge current densities (**c**) and cycling performance at a current density of 12 A g^−1^ (**d**) of the MS-Ni hollow nanostructures. The inset in d shows the first three charge-discharge curves at the same current density.

**Figure 6:**
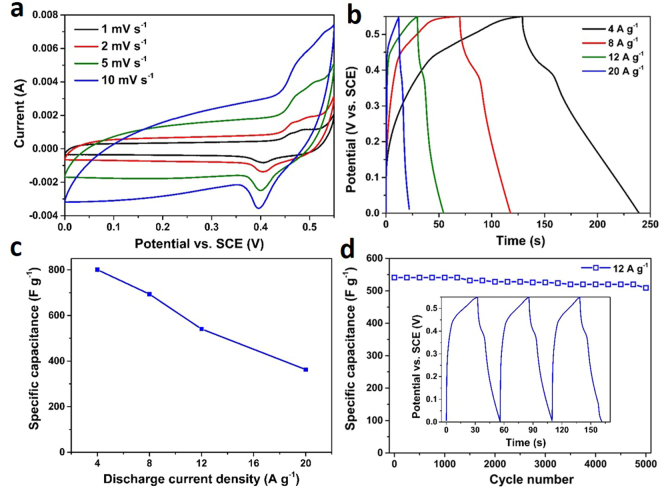
CV curves (**a**), GCCD curves (**b**), specific capacitances calculated from different discharge current densities (**c**) and cycling performance at a current density of 12 A g^−1^ (**d**) of the MS-Cu hollow nanostructures. The inset in d shows the first three charge-discharge curves at the same current density.

**Figure 7:**
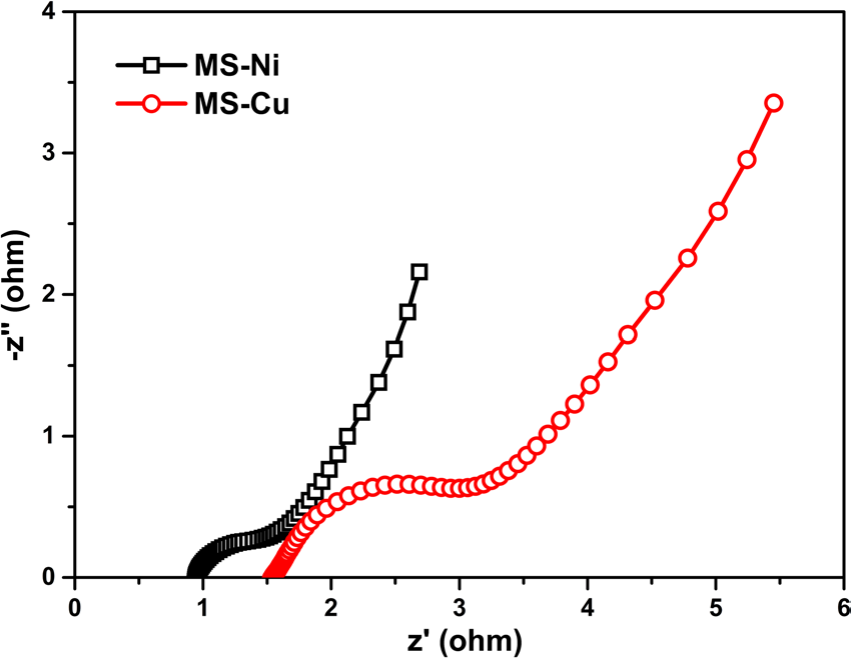
EIS curves of the MS-Ni and MS-Cu hollow nanospheres.

The electrochemical performances of the MS samples were further tested by different CV scan rates with a potential range of 0~0.55 V (vs. SCE). Figure 5a presents the CV results of the MS-Ni hollow spheres, where a pair of redox peaks can be distinctly observed, indicating a high electroactivity of the MS-Ni material^42^. The Galvano static charge-discharge (GCCD) measurements were then conducted at different current densities (4–20 A g^−1^) for the same sample (Fig. 5b), where the observed potential plateaus is associated with the redox peaks in CV. As a result, high specific capacitances of 1460, 1218, 1047, and 727 F g^−1^ can be calculated from GCCD curves at current densities of 4, 8, 12, and 20 A g^−1^, respectively (Fig. 5c). It should be noted that part of the calculated capacitance from GCCD is contributed by the non-Faradaic potential ranges, where a double-layer mechanism was involved for the charge storage. Thus, the MS-Ni material herein serves as an electrode for a hybrid supercapacitor, which employs both double-layer capacitance and Faradaic capacitance for the charge storage^43^. Cycling stability is considered very important for hybrid supercapacitors. Then, the current density of 12 A g^−1^ was chosen to evaluate the cycling stability of the MS-Ni electrode (Fig. 5d, the inset shows the first three GCCD cycles). After 5000 cycling times, the specific capacitance can be maintained at 980 F g^−1^, indicating a good capacitance retention of 93.6%. The electrochemical performance of this type of Ni_3_S_2_ hollow spheres is comparable to many of the reported work^19,26,44–46^. Next, the capacitive property of the MS-Cu sample was also evaluated by a similar measurement. The CV and GCCD curves are exhibited in Fig. 6a,b. Lower capacitance of 801, 694, 541, and 363 F g^−1^ are obtained at current densities of 4, 8, 12, and 20 A g^−1^, respectively (Fig. 6c). At a current density of 12 A g^−1^, this MS-Cu electrode was also cycled for 5000 times and a capacitance of 509 F g^−1^ can be retained with a capacitance retention of 94%, as shown in Fig. 6d. When compared to some of the previous work of CuS based materials^47–49^, the MS-Cu hollow structures herein also show exciting performances, demonstrating their potential applications as electrodes for next-generation high-performance hybrid supercapacitors. The electrochemical impedance spectroscopy (EIS) of the MS hollow nanospheres (MS-Ni and MS-Cu) was carried out from 100 kHz to 1 Hz with an amplitude of 5 mV, and the Nyquist plots of the two samples are presented in Fig. 7. A smaller semicircle in the high frequency range together with the smaller intercept on X axis has shown a higher electric conductivity of the MS-Ni sample, which again verified the better supercapacitor performance of MS-Ni than that of MS-Cu. These enhanced specific capacitances and good cycling performances of the MS hollow structures could be attributed to high electroactivity, unique hierarchical ultrathin shells and large specific surface area^50^. Some of the relevant work on nickel sulfides or copper sulfides for supercapacitors reported in recent years have been listed and compared with the results in this work (Table 1), which shows that the capacitance and cycling stability of our materials are comparable to some of the previous reports.

**Table 1:** A comparison of supercapacitor performances of nickel/copper sulfide based electrodes in previous work.

Active material	Substrate	Electrolyte	Highest specific capacitance (F g^−1^)	Cycling performance	Reference
Ni_3_S_2_ hollow spheres	Ni foam	1 M KOH	1460	93.6% after 5000 cycles	This work
CuS/Cu_1.8_S hollow spheres	Ni foam	1 M KOH	801	94% after 5000 cycles	This work
C@Ni_3_S_2_@MoS_2_ double core–shell nanorods	Ni foam	6 M KOH	1544	92.8% after 2000 cycles	J. Mater. Chem. A, 2016, 4, 1319–1325
3D CoNi_2_S_4_-graphene-2D-MoSe_2_ nanocomposite	Ni foam	6 M KOH	1141	108% after 2000 cycles	Adv. Energy Mater. 2016, 1600341
Ni_3_S_2_ nanosheet arrays	Ni foam	1 M NaOH	1300	76.9% after 20000 cycles	ACS Appl. Mater. Interfaces 2017, 9, 496−504
N_i3_S_2_ nanosheets	Ni foam	2 M KOH	N.A.	93.6% after 3000 cycles	RSC Adv., 2015, 5, 25446–25449
3D N_i3_S_2_ nanosheet arrays	Ni foam	6 M KOH	1370	91.6% after 5000 cycles	RSC Adv., 2015, 5, 32976–32982.
Ni_3_S_2_ nanorods@Ni(OH)_2_	Ni foam	3 M KOH	1277	99.1% after 2000 cycles	Energy Environ. Sci., 2013, 6, 2216–2221
CuS nanosheets	Ni foam	6 M KOH	833	75.4% after 500 cycles	J. Alloys Comp., 2015 625, 158–163
Double-shell CuS nanocages	Ni foam	2 M KOH	843	89.2% after 4000 cycles	J. Power Sources, 2017, 355, 31e35
flower-like CuS	Glassy carbon	2 M KOH	597	80% after 1000 cycles	Mater. Lett., 2014, 122, 25–28

**Figure 8:**
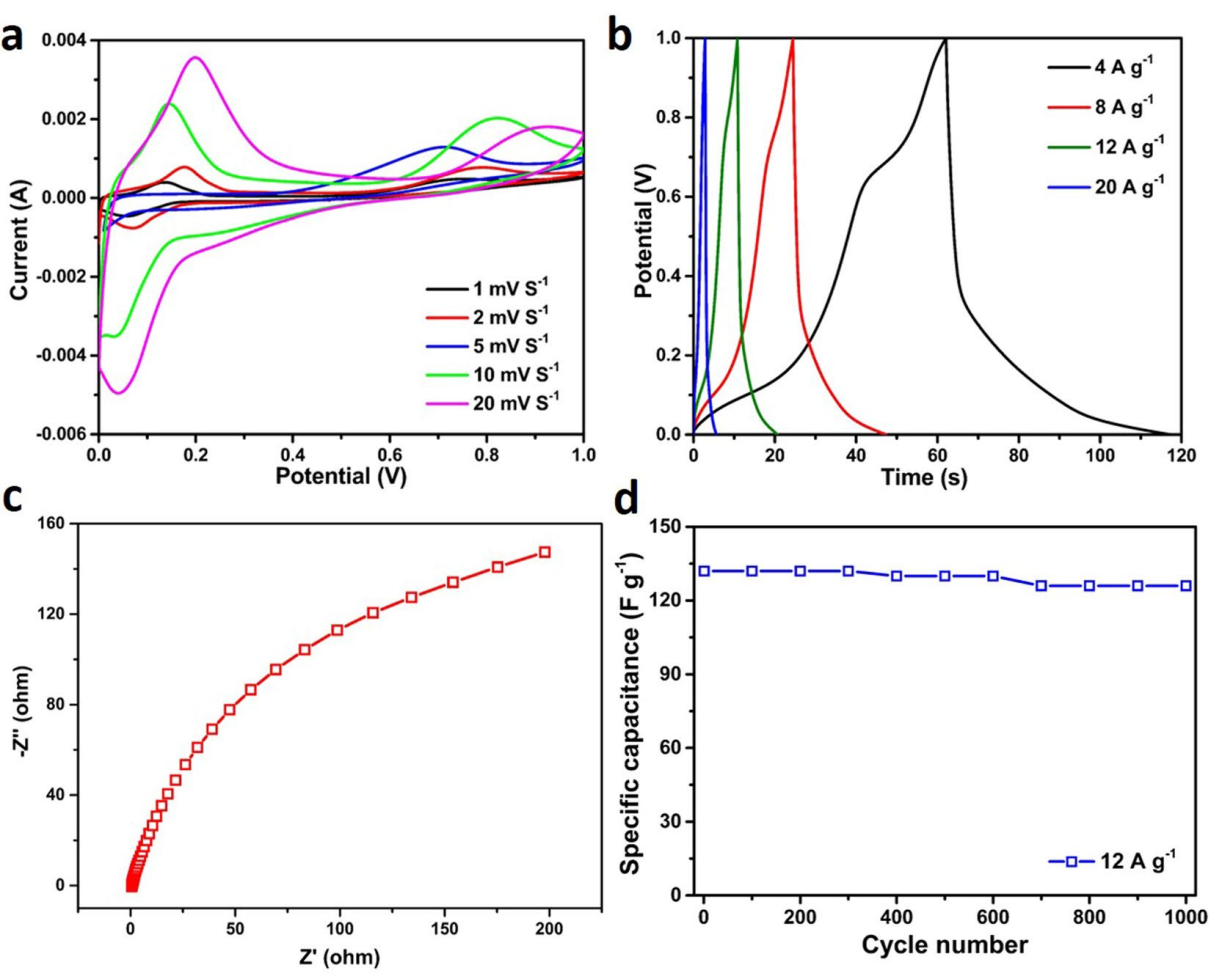
CV (**a**), GCCD (**b**), EIS (**c**) curves and cycling performance (**d**) of the asymmetrical supercapacitors fabricated from MS-Ni and MS-Cu hollow nanospheres.

To further evaluate the electrochemical performances of the as-prepared MS hollow nanospheres, asymmetrical supercapacitors (ASC) using the MS-Ni sample as the cathode and MS-Cu sample as the anode were assembled for testing. The CV measurements were performed at different scan rates within a potential range of 0–1 V, in which distinct redox peaks can be observed clearly, indicating the high electroactivity of the electrode materials (Fig. 8a). The subsequent GCCD tests (Fig. 8b) were conducted at different current densities, and high specific capacitances of 224, 192, 132 and 56 F g^−1^ can be calculated from the full cells at 4, 8, 12 and 20 A g^−1^, respectively. The EIS result shown in Fig. 8c reveals a higher impedance compared to the results of three-electrode system (Fig. 7), which could be one of the reasons for lower capacitance of two-electrode system. The cycling performance of the full cell was also evaluated at a high current density of 12 A g^−1^ and the result is present in Fig. 8d. The ASC delivered an initial specific capacitance of 132 F g^−1^ and 95.4% of the capacitance can be retained after 1000 cycles, showing a good cycling stability of the full cell device.

In summary, we report the fabrication of metal sulfide hollow nanospheres with hierarchical subunits through a facile template-engaged method. Hierarchical nanosheets of nickel sulfide and hairy nanoneedles of copper sulfide have been prepared from different metal based precursor, respectively. The as-prepared MS hierarchical hollow particles exhibit well-defined hollow structure with high uniformity, large surface area and excellent structural stability. By virtue of these advantageous features, these MS electrode materials manifest high specific capacitances(up to 1460 F g^−1^ and 224 F g^−1^ in ASC) with good cycling stability (up to 94% retention after 5000 cycles in three-electrode system and 95.4% in ASC), demonstrating their great potential in high-performance supercapacitors.

## Electronic supplementary material

Revised supplementary files
