# Supplementary material for: Fast ion transport through ultrathin shells of metal sulfide hollow nanocolloids used for high-performance energy storage
Source: Sci Rep. 2018 Jan 8;8:30. doi: 10.1038/s41598-017-18504-6 (PMC5758753; doi:10.1038/s41598-017-18504-6)
Supplement: Supplementary file 1 — Revised supplementary files [file 41598_2017_18504_MOESM1_ESM.doc]

**Electronic Supplementary Information**

**Fast Ion Transport through Ultrathin Shells of Metal Sulfide Hollow Nanocolloids Used for High-performance Energy Storage**

Zhenhua Chen^1,*^, Mengen Zhao^1^, Xinyan Lv^1^, Kang Zhou^1^, Xiaoqian Jiang^1^, Xiuli Ren^1,*^, and Xifan Mei^1,*^

^1^Jinzhou Medical University, Jinzhou, 121001, People’s Republic of China

^*^Corresponding Authors, (Z. C.) [zhchen561@yahoo.com](mailto:zhchen561@yahoo.com);

(X. R.) [rxlrenxiuli@163.com](mailto:rxlrenxiuli@163.com); (X. M) [meixifan1971@163.com](mailto:meixifan1971@163.com)


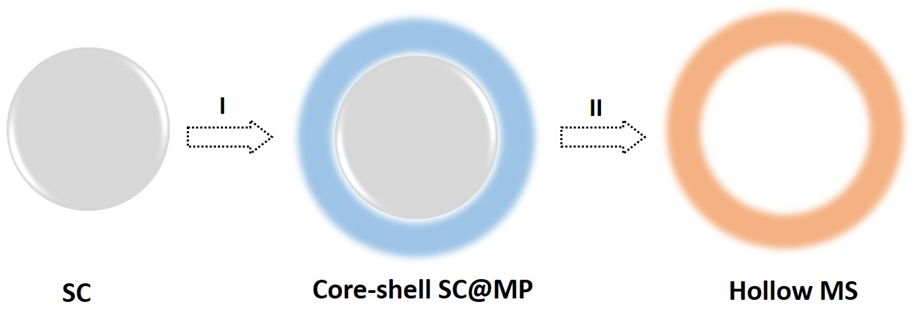


**Fig. S1** Schematic illustration of the formation of MS hollow nanostructures by a template-engaged method: MP deposition against silica colloids (I); MP converted to MS hollow nanostructures by a hydrothermal sulfurization (II).


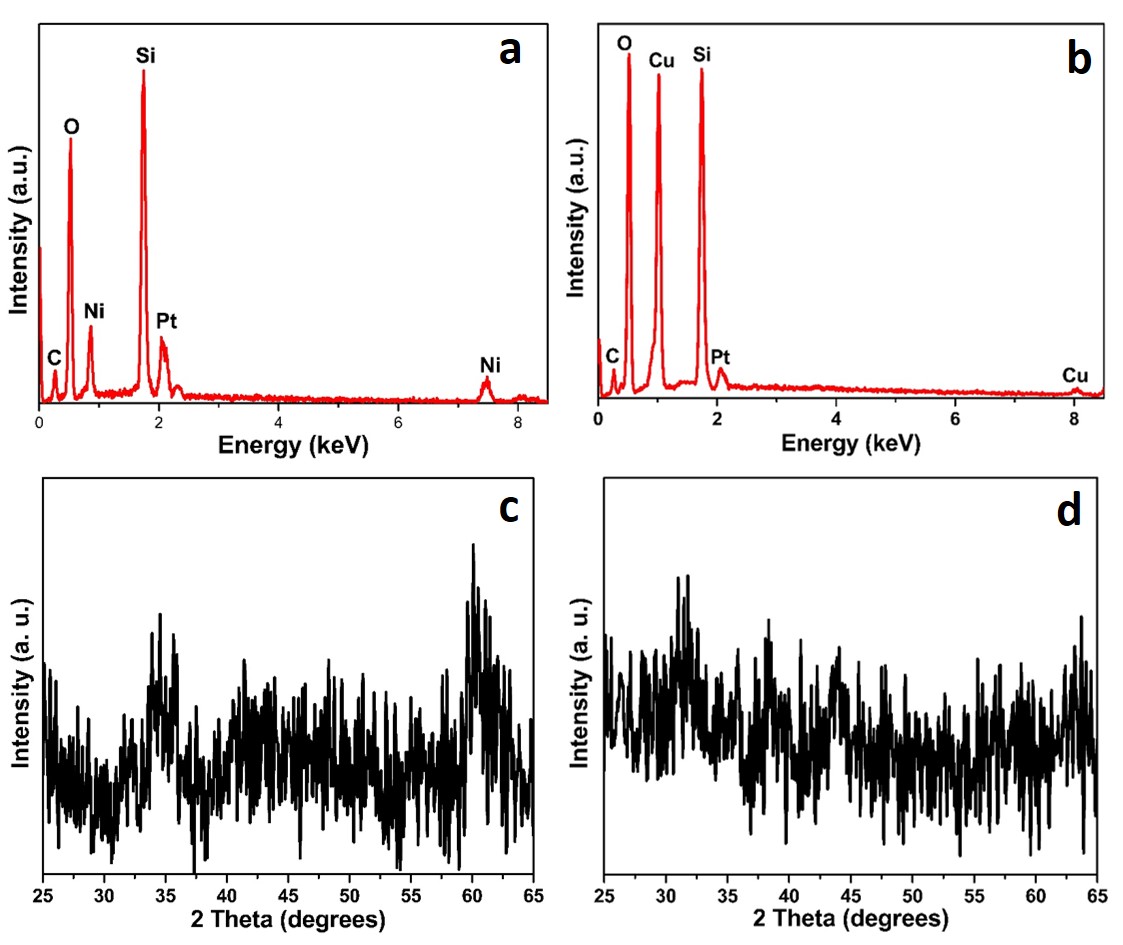


**Fig. S2** EDX (a and b) and XRD patterns (c and d) of the as-prepared MP-Ni (a and c) and MP-Cu (b and d).





**Fig. S3** EDX patterns of the as-formed MS-Ni (I, spheres), MS-Ni (II, worms), MS-Cu (III, spheres) and MS-Cu (IV, worms) hollow nanostructures.


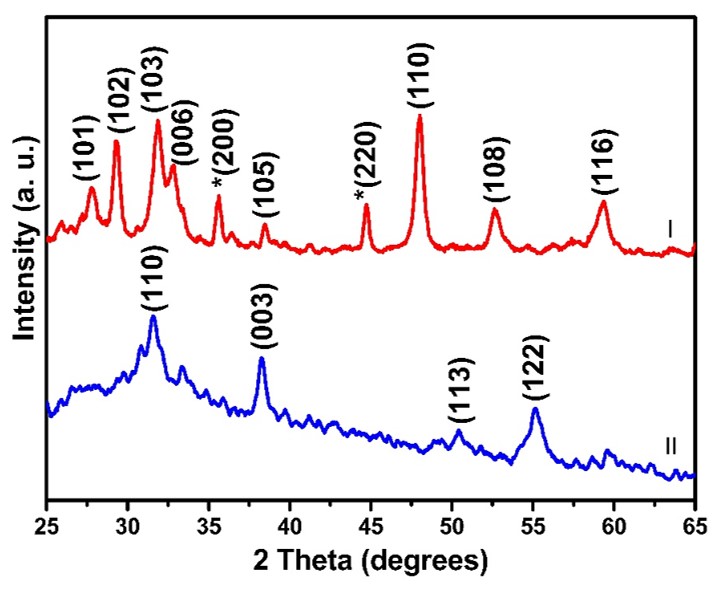


**Fig. S4** XRD patterns of the as-formed MS-Cu (I) and MS-Ni (II) hollow nanostructures.


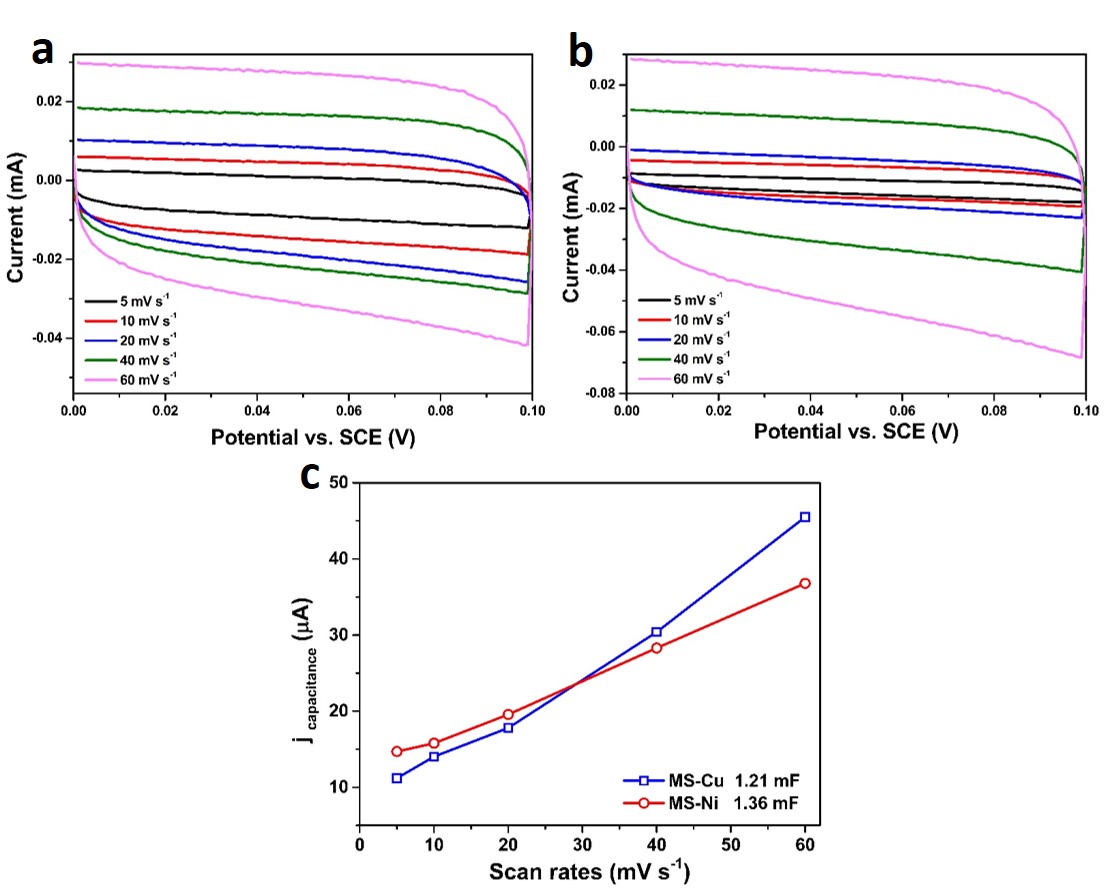


**Fig. S5** CV curves (a and b) and capacitance estimated from different CV scan rates (c) of the MS-Ni and MS-Cu electrodes.
